# Surface Treatment on Physical Properties and Biocompatibility of Orthodontic Power Chains

**DOI:** 10.1155/2017/6343724

**Published:** 2017-04-30

**Authors:** H. C. Cheng, M. S. Chen, B. Y. Peng, W. T. Lin, Y. K. Shen, Y. H. Wang

**Affiliations:** ^1^School of Dentistry, College of Oral Medicine, Taipei Medical University, Taipei, Taiwan; ^2^Department of Dentistry, Taipei Medical University Hospital, Taipei, Taiwan; ^3^School of Oral Hygiene, College of Oral Medicine, Taipei Medical University, Taipei, Taiwan; ^4^School of Dental Technology, College of Oral Medicine, Taipei Medical University, Taipei, Taiwan; ^5^Department of Mechanical Engineering, National Taiwan University of Science and Technology, Taipei, Taiwan

## Abstract

The conventional orthodontic power chain, often composed of polymer materials, has drawbacks such as a reduction of elasticity owing to water absorption as well as surface discoloration and staining resulting from food or beverages consumed by the patient. The goal of this study was to develop a surface treatment (nanoimprinting) for orthodontic power chains and to alleviate their shortcomings. A concave template (anodic alumina) was manufactured by anodization process using pure aluminum substrate by employing the nanoimprinting process. Convex nanopillars were fabricated on the surface of orthodontic power chains, resulting in surface treatment. Distinct parameters of the nanoimprinting process (e.g., imprinting temperature, imprinting pressure, imprinting time, and demolding temperature) were used to fabricate nanopillars on the surface of orthodontic power chains. The results of this study showed that the contact angle of the power chains became larger after surface treatment. In addition, the power chains changed from hydrophilic to hydrophobic. The power chain before surface treatment without water absorption had a water absorption rate of approximately 4%, whereas a modified chain had a water absorption rate of approximately 2%–4%. Furthermore, the color adhesion of the orthodontic power chains after surface modification was less than that before surface modification.

## 1. Introduction

Orthodontic power chains have been widely used in clinical orthodontic treatment since the 1960s [1] and are the most commonly used instrument for moving teeth. They are primarily composed of polyesters or polyethers formed through the polymerization of rubbers with multiple molecular structures connected by a series of urethane bonds [–(NH)–(CO)–O–] [2]. In clinical orthodontic treatment, the highly flexible nature of orthodontic power chains facilitates closing extraction spaces and adjusting the gear shaft angle [3]. Orthodontic power chains have many clinical advantages. Specifically, they are economic, are both easy to use and easy to adjust to the patient's needs, and provide light, continuous, and powerful assistance in appropriate tooth movement. However, because of the natural instability of the rubber, they are susceptible to the effects of changes in their surrounding environment. Therefore, the shape and size of orthodontic force affect the efficiency of orthodontic treatment.

The impact strength of orthodontic power chains is affected by intrinsic factors such as the material composition, production methods, and external morphology [4, 5] and external environmental factors such as temperature, ambient pH, and moisture absorption levels. These factors all require correction of the power chains so that they can maintain strength and provide permanent deformation. In particular, because the oral cavity environment is extremely humid and prone to dramatic changes in temperature, it is difficult for corrective rubber chains to provide a continuous and stable force, preventing orthodontists from accurately measuring the force exerted on the teeth and in turn causing the number of patients returning to the clinic to increase [6]. Because water molecules are plasticizers, they weaken the interaction between molecules, resulting in chemical degradation [7]. Immersing a rubber chain in water for 7 days revealed that ester and ether molecules were leached because hydrolysis destroyed the backbone of the molecular chains [8].

Anodic aluminum oxide (AAO) films were fabricated through anodization of pure aluminum (Al) in suitable electrolytic solutions. These AAO films were manufactured with many nanopores, and the pore diameter and cell size, measured as the distance between the centers of two neighboring pores, were controlled by applying a pore-widening treatment, in which pores were etched chemically [9, 10]. Many researchers have achieved patterning using a nanoporous AAO membrane as a mask on injection molding or imprinting, using cycloolefin copolymer (COC) and polyvinyl chloride (PVC) as molded materials. The contact angles of smooth surfaces of COC and PVC were about 89°–90°. The contact angles of microbumps and nanopillars (COC) were 120°–140°. The surface of COC material on which the nanostructures were superimposed on the microstructures appeared hydrophobic [11]. Anodization yielded porous metallic membranes on nanoimprinting. Tapered holes were manufactured through repeated anodization by a pore-widening method. A polymer surface was then achieved by filling the tapered holes. This polymer with this unique surface geometry had antireflective properties [12].

Many microplastic imprinting processes can be applied to micropatterns including microdispensing combined with a hot imprinting process [13], UV molding [14], hybrid extrusion rolling imprinting [15], gas-assisted imprinting [16], and microelectrical discharge machining combined with a hot imprinting process [17, 18]. The nanoimprinting process has attracted attention from fabricating because it is applied to mass-produce nanostructure products at low costs and high production rates.

The target of this study was to identify an effective and rapid mass-production method on the surface treatment of orthodontic power chains. In this study, a concave, nanoporous AAO film was adopted as a template for nanoimprinting to fabricate on an orthodontic power chain with a convex nanopillar. The objective of this study was to improve the surface properties and reduce water absorption and color stains on the orthodontic power chain.

## 2. Experimental

### 2.1. AAO Template Fabrication

An AAO template (concave) with localized conical pores was fabricated through repeated anodization and pore-widening method, among which the two anodization procedures were alternated. Each anodization step was kept short to fabricate cones with low aspect ratios (cone height eliminated by base diameter of cone). A conical AAO template with a low aspect ratio was to manufacture some functional nanomaterials. This template can be applied in electrochemical deposition, sol-gel dipping, or an evaporation process. The low aspect ratio of pores causes simple material deposition. The materials tend to be deposited at the pore's top, such that the pore's bottom is not filled. This problem is exacerbated as the pore's aspect ratio increases. A 99.99% pure aluminum film (100 × 20 × 10 mm^3^; Wako Pure Chemical Industry, Ltd., Taiwan) was electrochemically polished in a solution of 60% HClO_4_ and 95% C_2_H_5_OH at a ratio of 1 : 4. This film was cleaned by ethanol and pure water and then took advantage of manufacturing the conical AAO template. The film and a carbon electrode acted as the anode and cathode, respectively. The aluminum was anodized at 60 V in a 0.3 M oxalic acid solution at 20°C. To manufacture the AAO template, anodization for 1 h was used to produce a hexagonally ordered pore array. The AAO template was dissolved in a solution of 1.5 wt% chromic acid and 6 vol% phosphoric acid. To manufacture conical pores in this template, anodization and pore-widening treatments were alternated. The template was then anodized employing the same solution and voltage to fabricate uniformly sized pores. The pores were then widened through chemical etching, and the template was once again anodized under the same anodization operating conditions. At this time, the pores formed the taper; the interior of each pore comprised a two-step structure. To obtain the desired inverted conical structure, each step was fulfilled twice in the anodization and pore-widening process. Each anodization step was conducted at temperature of 9°C and voltage of 60 V in the same solution. The anodization time was 25 seconds in the first step and 20 seconds in each subsequent step. During the pore-widening treatment, the specimen was dipped in a 5 vol% phosphoric acid solution at 30°C for 12 minutes. The experimental results showed that the mean diameter of the nanoholes of the AAO was 200 nm at a voltage of 60 V for anodized oxidation.

### 2.2. Scanning Electron Microscopy and Atomic Force Microscopy for AAO

The specimen morphology was viewed by the way of field emission scanning electron microscopy (SEM) (JSM-6700F; JOEL, Japan). Cross sections of the AAO templates were intended by bending the aluminum until the template fractured. The AAO specimen was coated with platinum through sputtering prior to observations. The detailed morphology of specimen was observed utilizing atomic force microscopy (AFM) (Nanosurf Mobile S; Swiss). Prior to replication, the AAO template self-assembled to comprise an antiadhesive monolayer (1H, 1H, 2H, and 2H-perfluorodecyltrichlorosilane) through vapor phase deposition and minimize the surface energy of AAO template, the latter of which is required for easy demolding of the power chain from the AAO template.

### 2.3. Nanoimprinting on Power Chains

**Figure 1:**
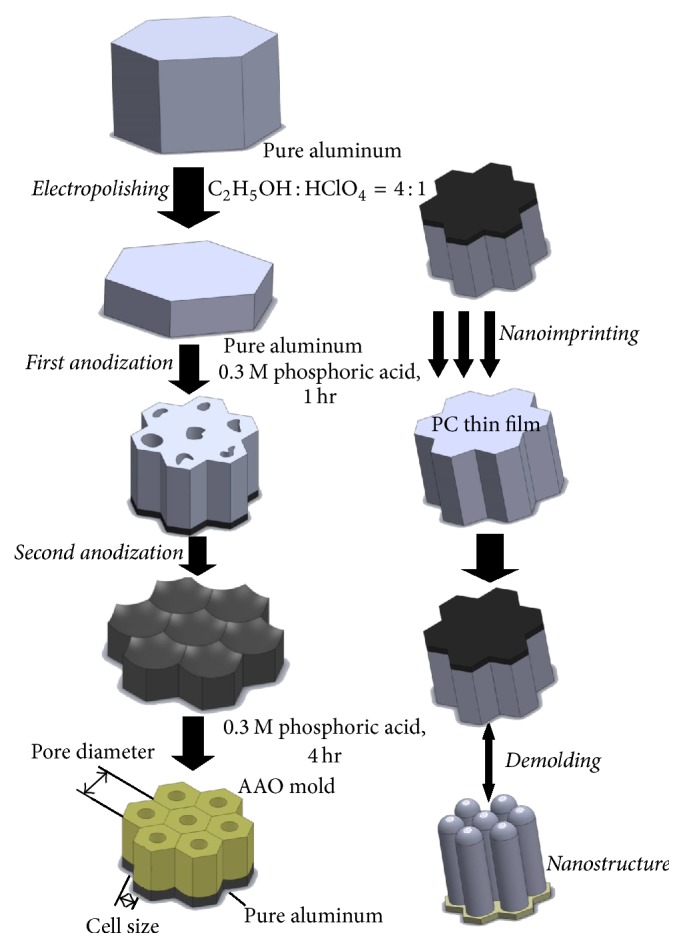
Complete process for AAO template fabrication and nanoimprinting.

**Figure 2:**
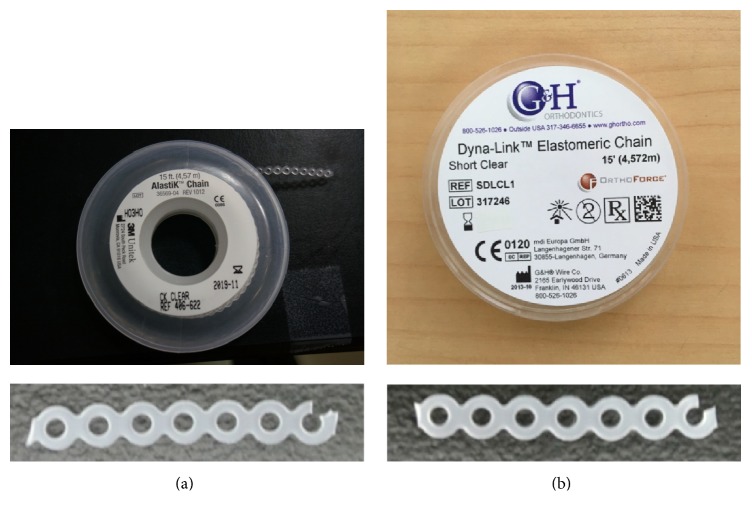
Orthodontic power chains. (a) 3M Alastik chain, (b) Dyna-link chain.

**Figure 3:**
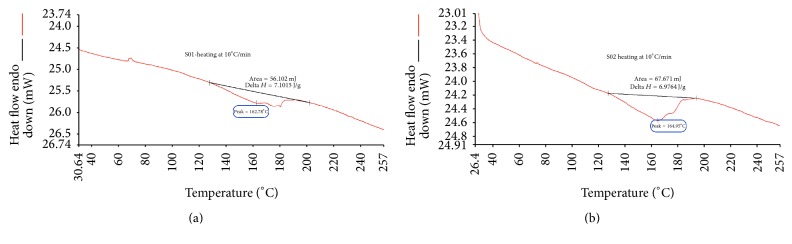
Glass transition temperatures of different power chains. (a) 3M Alastik chain, (b) Dyna-link chain.

After the AAO template was fabricated, a nanoimprinting machine (NIL-3.0 Imprinter; Obduct AB, Sweden) fulfilled imprinting.  Figure 1 indicates the process from AAO template fabrication to nanopillar fabrication of power chain. Elastomeric power chains (3M Alastik Chain, Dyna-link elastomeric chain) were applied as molded materials during nanoimprinting (Figure 2). The 3M Alastik chain was transparent and the Dyna-link chain was gray. A differential scanning calorimeter (DSC, DSC 400, Perkin Elmer) was applied to measure the glass transition temperatures of the elastomeric power chains. The results revealed that the glass transition temperature was 162.78°C for the 3M Alastik chain and 164.95°C for the Dyna-link chain (Figure 3). These results facilitated setting the parameters on the nanoimprinting process, namely, the imprinting temperature, pressure, time, and demolding temperature (Table 1).

### 2.4. Surface Properties of Power Chains

A contact angle meter (DGD-DI; Digidrop Ltd., France) was applied to measure the contact angle of the orthodontic power chains before and after surface treatment. Each specimen was measured at 5 points. Deionized (DI) water in 0.5 *μ*L drops was administered on the sample surface. Subject to a drop in solid/gas/liquid 3-state stability, computer-controlled photography (25/s) was applied to capture images and convert the image files. Measured contact angle data were obtained and plotted in charts. Water absorption necessitates correction of power chains. Consequently, the performance of the nanoimprinted power chains after absorption correction was measured. The power chains were corrected using a vacuum oven at 60°C and immersed in water for three days. The power chains were then weighed (*W*_dry_) and placed in 37 ± 2°C DI water for one day. Finally, they were removed and weighed (*W*_wet_) to calculate the absorption rate

(1) $$\begin{array}{c} Moisture=\frac{\left(W_{wet}-W_{dry}\right)}{W_{dry}}\times 100\%. \end{array}$$

The degree of staining was observed on this study. Experiments proceeded as follows:

1. The sample was placed in a vacuum oven at 60°C and immersed in water for three days.
2. The sample was then placed in 37°C test liquid (e.g., red ink, soy sauce).
3. After two days, the sample was washed with distilled water for one minute, and a digital camera was used to take pictures to visually compare staining and each imprinting parameter of five samples.

### 2.5. Statistics

In this study, measured data were subjected to statistical analysis. For any given experiment, each data point represented the mean ± standard deviation (SD) of six individual experiments. The *t*-test was used to determine significance between groups in the contact angle. Statistical significance was revealed by ^*∗*^*p* < 0.05, ^*∗∗*^*p* < 0.01.

## 3. Results and Discussion

### 3.1. Nanoholes of AAO

**Figure 4:**
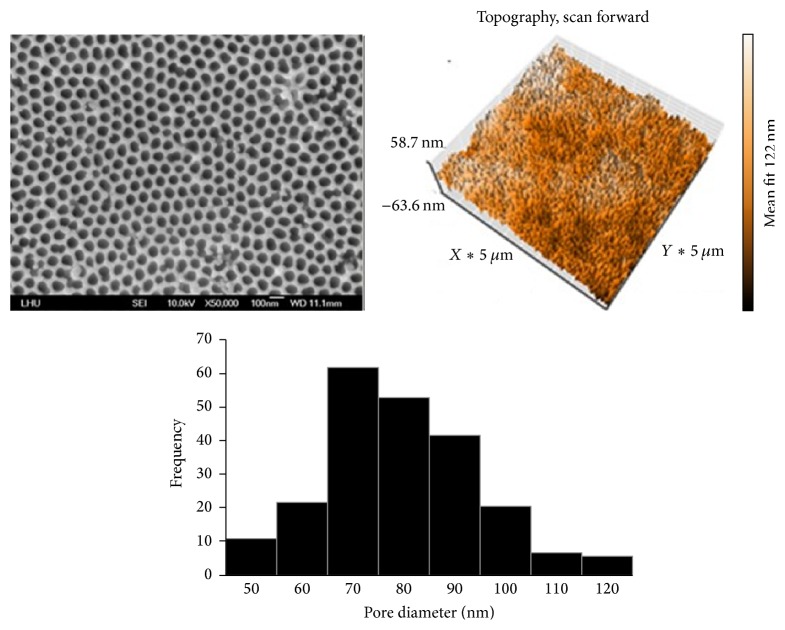
SEM images and surface roughness of the AAO (Φ = 100 nm, Ra = 10.83 nm).

**Figure 5:**
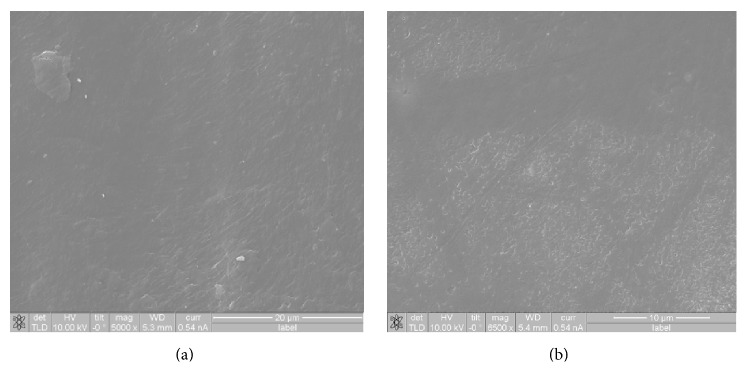
SEM images of different molded orthodontic power chains before nanoimprinting. (a) 3M Alastik chain, (b) Dyna-link chain.

Figure 4 reveals the nanoholes in the AAO template observed by SEM and AFM. The mean diameter of the nanoholes in the AAO was 200 nm. The nanoholes in the sample were of 24,000 nm depth. The results showed that the shape of the nanoholes was favorable for anodization and demonstrated that the nanoholes had a high aspect ratio of 120. According to SEM observation, the power chain had a smooth surface with no distinct structure (Figure 5).

### 3.2. Nanostructure of the Power Chains

**Figure 6:**
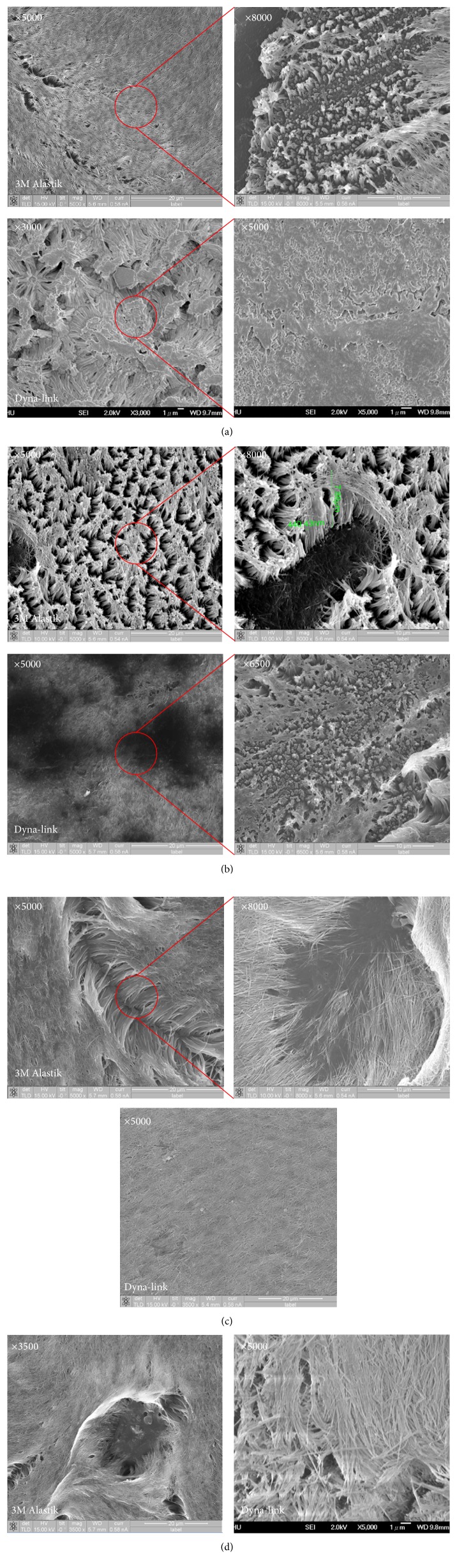
SEM images of nanopillars of different molded orthodontic power chains for nanoimprinting using the AAO template. (a) Nanoimprinting with processing condition A. (b) Nanoimprinting with processing condition B. (c) Nanoimprinting with processing condition C. (d) Nanoimprinting with processing condition D.

Figure 6 shows the nanostructure, containing convex nanopillars, of orthodontic power chains by applying the AAO template in nanoimprinting. Nanoimprinting fabricated favorable nanopillar shapes (Figure 6). The effects of nanoimprinting parameters on the surface properties of the different orthodontic power chains with and without nanopillars are discussed as follows. The power chain surface properties formed under processing condition A (an imprinting temperature of 155°C, an imprinting pressure of 50 bar, an imprinting time of 180 s, and a demolding temperature of 50°C) were observed using SEM at 5000-fold magnification. The fibrous nanostructures generated had incomplete forms, with their surfaces being homogeneous, their fibrous structures mixed, and the columnar nanopillars produced not evenly distributed (Figure 6(a)). Under 8000-fold magnification, then the rod fibers were more clearly observed, being shorter than those observed under condition A and of different heights and shapes, with longer nanorods sticking to each other at their tops. The surface properties of power chain formed under processing condition B (an imprinting temperature of 160°C, an imprinting pressure of 50 bar, an imprinting time of 180 s, and a demolding temperature of 50°C), applied in the manufacturing process, were observed by SEM at 5000-fold magnification. The formation of more completely uniform nanopillar columns was found, with the embossing height being higher than that for condition A. In addition, although a few of the top beam nanopillar columns adhered to each other, they still maintained a basic columnar structure and did not agglomerate into a sheet (Figure 6(b)).

### 3.3. Influence of Nanoimprinting Conditions on Nanostructure of Power Chain

According to observation under 8000-fold magnification and measurement of the nanopillar column diameter and height, this group of processing parameters yielded the most complete three-dimensional nanopillar column structures. The contact angles of the hydrophobic optimal set of parameter settings are used under the same condition; therefore, we applied it to measure the proportion of nanopillar columns with a diameter of approximately 440.43 nm, a height of approximately 7.88 *μ*m, and a diameter-to-height ratio of 1 : 17.89. A high diameter-to-height ratio results in a nanopillar column with a Van der Waals force vulnerable to the impact caused by columns adhering to each other, thus reducing the surface modification effectiveness (Figure 6(b)). The surface properties of power chain under processing condition C (an imprinting temperature of 165°C, an imprinting pressure of 50 bar, an imprinting time of 180 s, and a demolding temperature of 50°C) were observed by SEM at 5000-fold magnification. Elongated nanopillar columns were found. The results found that the nanofiber columns were gradually sticky into flakes, losing the original three-dimensional columnar appearance. It is found that the contact angle value decreases under this process parameter (Figure 6(c)). The same set of parameters was still clearly observed by SEM at 8000-fold magnification, but the fibers became more slender, increasing the diameter and height differences, and adhered to one another. The middle portion of vacancies was presumably caused by ejection defects (Figures 6(c) and 6(d)). According to SEM observation, stripping parameter settings have a substantial impact on results. As the temperature gradually exceeds the glass transition temperature of the sample, nanometer fiber columns easily agglomerate into a sheet. Improper stripping is likely to cause the entire film nanostructure to stall and the sample surface to become uneven, affecting the results of surface modification. The proposed technique provides effective demolding of the AAO template from the power chain through the use of an antiadhesive monolayer coated on the AAO template.

### 3.4. Surface Properties of Power Chains

**Figure 7:**
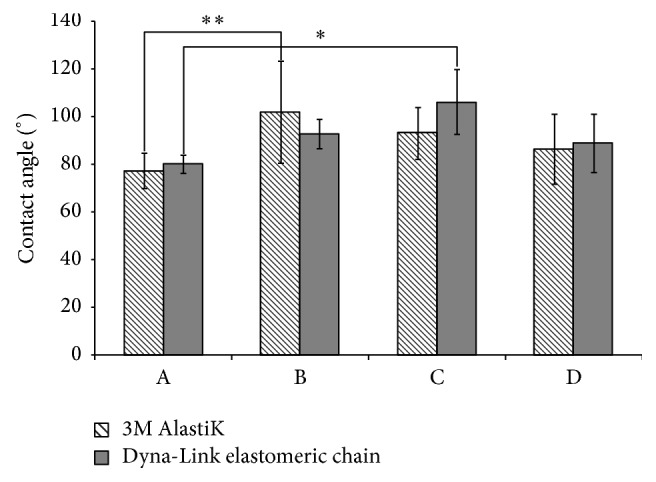
Contact angles of orthodontic power chains before and after surface treatment (values are the mean ± SD of six experiments (*n* = 6), ^*∗*^*p* < 0.05, ^*∗∗*^*p* < 0.01).

Figure 7 shows the contact angles of orthodontic power chains with and without nanopillar. The contact angles of power chains without surface modification were 67.4° (3M Alastik) and 66.2° (Dyna-link); these appear hydrophilic. Processing condition A entailed an imprinting temperature of 155°C; an imprinting pressure of 50 bar; an imprinting time of 180 s; and a demolding temperature of 50°C; the mean contact angles were 77.36° (3M Alastik) and 80.13° (Dyna-link), indicating that the chains were hydrophilic. Processing condition B entailed an imprinting temperature of 160°C; an imprinting pressure of 50 bar; an imprinting time of 180 s; and a demolding temperature of 50°C; the mean contact angles were 101.69° (3M Alastik) and 92.83° (Dyna-link), indicating that the chains were hydrophobic. Processing condition C entailed an imprinting temperature of 165°C; an imprinting pressure of 50 bar; an imprinting time of 180 s; and a demolding temperature of 50°C; the mean contact angles were 92.98° (3M Alastik) and 106.1° (Dyna-link), indicating that the chains were hydrophobic. Processing condition D comprised an imprinting temperature of 170°C; an imprinting pressure of 50 bar; an imprinting time of 180 s; and a demolding temperature of 50°C; the mean contact angles were 86.36° (3M Alastik) and 88.9° (Dyna-link), indicating that the chains were hydrophilic. The contact angles indicate the statistically significant difference among processing conditions A and B for 3M Alastik power chain. The results also reveal the statistically significant difference between processing conditions A and C for Dyna-link power chain.  Table 2 lists the absorbance rates of different orthodontic power chains after nanoimprinting. The weights of power chains were 0.005 g (3M Alastik) and 0.004 g (Dyna-link) before the nanoimprinting process without water absorption and were 0.0052 g (3M Alastik) and 0.0042 g (Dyna-link) before the nanoimprinting process with water absorption. The water absorption rates were 4% (3M Alastik) and 5% (Dyna-link). The weights of the power chains were 0.0054 g (3M Alastik) and 0.004 g (Dyna-link) under processing condition A without water absorption and 0.0056 g (3M Alastik) and 0.0042 g (Dyna-link) under the same processing condition with water absorption. The water absorption rates were 3.7% (3M Alastik) and 5% (Dyna-link). The power chain weights were 0.0051 g (3M Alastik) and 0.0034 g (Dyna-link) under processing condition B without water absorption and 0.0052 g (3M Alastik) and 0.0036 g (Dyna-link) under the same processing condition with water absorption. The water absorption rates were 2% (3M Alastik) and 4.4% (Dyna-link). The weights of the power chains were 0.0047 g (3M Alastik) and 0.0047 g (Dyna-link) without water absorption under processing condition C and 0.0049 g (3M Alastik) and 0.0048 g (Dyna-link) with water absorption under the same processing condition. The absorption rates were 4.2% (3M Alastik) and 2.1% (Dyna-link). The weights of the power chains were 0.0049 g (3M Alastik) and 0.0044 g (Dyna-link) without water absorption under processing condition D and 0.0051 g (3M Alastik) and 0.0048 g (Dyna-link) with water absorption under the same processing condition. The absorption rates were 4% (3M Alastik) and 5.5% (Dyna-link).

### 3.5. Dyeing Tests of Power Chains

**Figure 8:**
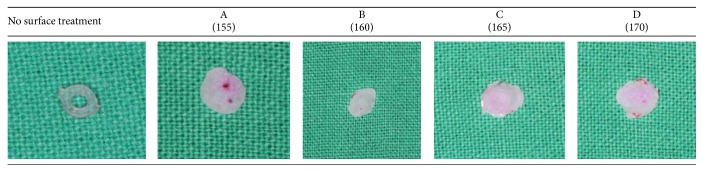
Dyeing test for orthodontic power chain (3M Alastik chain).

Figure 8 indicates the results of dyeing tests for orthodontic power chains (Figure 8). Because the Dyna-link elastomeric chain was gray and would not properly exhibit staining test results, we used only the 3M Alastik power chain for the dye tests. The specimens were dipped in red ink dye for 48 hours and are ranked from lowest to highest according to the staining depth, as determined by the naked eye, as follows: B < C < D = A. Differences among the other 4 groups are difficult to compare, but the naked eye falls on the color depth of the C and D specimens. The staining test results were generally in line with the contact angle measurements; however, accurate results regarding the color effect can be obtained by using a prolonged staining time and relevant computer equipment for further analysis.

## 4. Conclusions

In this study, an AAO template was applied as a mold insert for fabricating the nanostructures on orthodontic power chains by nanoimprinting. Experimental results revealed that the contact angle of the orthodontic power chains increased from approximately 80° to 130°. In addition, the surface property of power chain changed from hydrophilic to hydrophobic, and its absorption rate decreased after surface modification. In this investigation, nanopillars formed on the orthodontic power chains, and the power chain surface was modified using a high-speed mass-production process.

## Figures and Tables

**Table 1:** Levels of processing parameters for nanoimprinting.

	Parameter			
Condition	Imprinting temp. (°C)	Demolding temp. (°C)	Imprinting time (s)	Imprinting press. (bar)
A	155	50	180	50
B	160	50	180	50
C	165	50	180	50
D	170	50	180	50

**Table 2:** Absorption rates of orthodontic power chains before and after surface treatment (3M Alastik/Dyna-link).

	Weight before absorbing water (g)	Weight after absorbing water (g)	Weight difference (g)	Water absorption rate (%)
No surface modification	0.005/0.004	0.0052/0.0042	0.0002/0.0002	4%/5%
A	0.0054/0.004	0.0056/0.0042	0.0002/0.0002	3.7%/5%
B	0.0051/0.0034	0.0052/0.0036	0.0001/0.0002	2%/4.4%
C	0.0047/0.0047	0.0049/0.0048	0.0002/0.0001	4.2%/2.1%
D	0.0049/0.0044	0.0051/0.0048	0.0002/0.0004	4%/5.5%

## References

[1] Baty D. L., Storie D. J., von Fraunhofer J. A. (1994). Synthetic elastomeric chains: a literature review. *American Journal of Orthodontics and Dentofacial Orthopedics*.

[2] Billmeyer F. W. (1984). Thermosetting resins. *Textbook of Polymer Science*.

[3] Park Y.-C., Choi Y.-J., Choi N.-C., Lee J.-S. (2007). Esthetic segmental retraction of maxillary anterior teeth with a palatal appliance and orthodontic mini-implants. *American Journal of Orthodontics and Dentofacial Orthopedics*.

[4] Ferriter J. P., Meyers C. E., Lorton L. (1990). The effect of hydrogen ion concentration on the force-degradation rate of orthodontic polyurethane chain elastics. *American Journal of Orthodontics and Dentofacial Orthopedics*.

[5] Eliades T., Eliades G., Watts D. C. (1999). Structural conformation of in vitro and in vivo aged orthodontic elastomeric modules. *The European Journal of Orthodontics*.

[6] Brooks D. G., Hershey H. G. (1976). Effects of heat and time on stretched plastic orthodontic modules. *Journal of Dental Research*.

[7] Huget E. F., Patrick K. S., Nunez L. J. (1990). Observations on the elastic behavior of a synthetic orthodontic elastomer. *Journal of Dental Research*.

[8] Schollenberger C. S., Stewart F. D. (1971). Thermoplastic polyurethane hydrolysis stability. *Journal of Elastomers and Plastics*.

[9] Yuan J. H., He F. Y., Sun D. C., Xia X. H. (2004). A simple method for preparation of through-hole porous anodic alumina membrane. *Chemistry of Materials*.

[10] Wang X., Han G. R. (2003). Fabrication and characterization of anodic aluminum oxide template. *Microelectronic Engineering*.

[11] Koponen H.-K., Saarikoski I., Korhonen T. (2007). Modification of cycloolefin copolymer and poly(vinyl chloride) surfaces by superimposition of nano- and microstructures. *Applied Surface Science*.

[12] Masuda H. (2005). Highly ordered nanohole arrays in anodic porous alumina. *Ordered Porous Nanostructures and Applications*.

[13] Cheng H.-C., Wang C.-H., Huang C.-F. (2010). Micro fabrication of microlens arrays by micro dispensing. *Polymers for Advanced Technologies*.

[14] Kim S.-M., Kang S. (2003). Replication qualities and optical properties of UV-moulded microlens arrays. *Journal of Physics D: Applied Physics*.

[15] Jiang L.-T., Huang T.-C., Chiu C.-R., Chang C.-Y., Yang S.-Y. (2007). Fabrication of plastic microlens arrays using hybrid extrusion rolling embossing with a metallic cylinder mold fabricated using dry film resist. *Optics Express*.

[16] Huang T.-C., Chan B.-D., Ciou J.-K., Yang S.-Y. (2009). Fabrication of microlens arrays using a CO_2_-assisted embossing technique. *Journal of Micromechanics and Microengineering*.

[17] Huang M.-S., Chiang Y.-C., Lin S.-C. (2012). Fabrication of microfluidic chip using micro-hot embossing with micro electrical discharge machining mold. *Polymers for Advanced Technologies*.

[18] Huang C.-F., Cheng H.-C., Lin Y., Wu C.-W., Shen Y.-K. (2014). Study on cellar behaviors on different nanostructures by nanoporous alumina template. *International Journal of Precision Engineering and Manufacturing*.

